# MM-129 Counteracts 5-Fluorouracil-Induced Cellular Senescence in Colon Cancer via SIRT1/STAT3 Signaling Pathway

**DOI:** 10.3390/cells14191498

**Published:** 2025-09-24

**Authors:** Hubert Klepacki, Beata Sieklucka, Joanna Kalafut, Krystyna Kowalczuk, Arkadiusz Surazynski, Mariusz Mojzych, Anna Pryczynicz, Dariusz Pawlak, Natascia Tiso, Justyna Magdalena Hermanowicz

**Affiliations:** 1 Department of Pharmacodynamics, Medical University of Bialystok, Mickiewicza 2C, 15-222 Bialystok, Poland; dariusz.pawlak@umb.edu.pl (D.P.); justyna.hermanowicz@umb.edu.pl (J.M.H.); 2 Department of Monitored Pharmacotherapy, Medical University of Bialystok, 15-222 Bialystok, Poland; 3 Department of Biochemistry and Molecular Biology, Medical University of Lublin, Chodźki 1, 20-093 Lublin, Poland; 4 Department of Integrated Medical Care, Medical University of Bialystok, Mickiewicza 2C, 15-222 Bialystok, Poland; 5 Department of Medicinal Chemistry, Medical University of Bialystok, Mickiewicza 2C, 15-222 Bialystok, Poland; 6 Faculty of Health Science, Collegium Medicum, The Mazovian Academy in Plock, Plac Dabrowskiego 2, 09-402 Plock, Poland; 7 Department of General Pathomorphology, Medical University of Bialystok, 15-222 Bialystok, Poland; 8 Department of Biology, University of Padova, 35131 Padova, Italy; natascia.tiso@unipd.it; 9 Department of Clinical Pharmacy, Medical University of Bialystok, Mickiewicza 2C, 15-222 Bialystok, Poland

**Keywords:** colorectal cancer, cellular senescence, chemoresistance, MM-129, senescence-associated secretory phenotype, 5-fluorouracil, SIRT1/STAT3 signaling, senotherapeutic agent

## Abstract

Cellular senescence plays a critical role in tumorigenesis and is recognized as a hallmark of colorectal cancer (CRC). Emerging evidence suggests that 5-fluorouracil (5-FU)-induced senescence may contribute to chemoresistance and tumor recurrence. Here, we investigated the effect of 5-FU on colon cancer cell senescence and whether MM-129 (pyrazolo[4,3-e]tetrazolo[4,5-b][1,2,4]triazine sulfonamide) can antagonize this activity. Senescence was identified by the expression of senescence-associated β-galactosidase (SA-β-gal) and cyclin-dependent kinase inhibitor 1A (p21) using qPCR, microscopy, flow cytometry, and immunohistochemistry. We also measured interleukin 6 (IL-6) and tumor necrosis factor (TNF-α) as key SASP cytokines, along with E-cadherin (CDH1), a marker of epithelial integrity. The SIRT1/STAT3 pathway was evaluated to elucidate the mechanism of MM-129′s action. MM-129 counteracted 5-FU-induced senescence in colon cancer models, reducing p21 levels in zebrafish xenografts and the number of SA-β-gal-positive cells *in vitro* and in tumor tissues from DLD-1 and HT-29 mouse xenografts. MM-129 also inhibited senescence-associated responses by suppressing SASP cytokines (IL-6, TNF-α) and restoring E-cadherin (CDH1), and it modulated the SIRT1/STAT3 axis, which may underlie the observed senotherapeutic effects. In conclusion, MM-129 represents a novel senotherapeutic candidate. By modulating the SIRT1/STAT3 axis, it may suppress the SASP and weaken pro-survival signaling, thereby facilitating selective clearance of senescent cells. Integrating senotherapeutics with conventional cancer therapies may enhance efficacy and open new avenues for translational research.

## 1. Introduction

Senescent cells (SnCs) are cells that have stopped dividing and are no longer able to proliferate. There are two types of cellular senescence: replicative senescence related to telomere shortening and telomere-independent stress-induced premature senescence (SIPS) [1,2]. Replicative senescence results from the exhaustion of the division limit, while SIPS can be induced by oxidative stress, oncogenes or DNA-damaging agents, e.g., chemotherapeutics, ionizing radiation or other cytostatic compounds, termed therapy-induced senescence (TIS) [3]. Senescence cells can have both beneficial and detrimental effects depending on the physiological or pathological context. SnCs can play a role in wound healing by releasing factors that promote tissue repair. Demaria et al. observed that the removal of SnCs during wound healing delayed tissue regeneration in a mouse model [4]. In turn, an *in vivo* model of oncogene-induced senescence (OIS) demonstrated accelerated repair of locally transplanted keratinocytes transiently exposed to senescence-associated secretory phenotype (SASP) factors [5]. SASP can also have positive effects, such as inhibiting immune reactions in damaged cells or preventing fibrosis during the healing process [6,7]. However, senescence has dual role. While it has previously been described solely as a tumor-suppressive mechanism, SnCs can alternatively even activate carcinogenicity through SASP. This phenotype secretes pro-inflammatory cytokines, epithelial growth factors, and tissue remodeling enzymes that can promote the growth and survival of cancer cells [8,9,10]. They may create a supportive microenvironment for cancer cells to grow and spread. It has also been observed that the SASP can cause an epithelial–mesenchymal transition (EMT) phenomenon that promotes cancer [9,11,12].

Colorectal cancer (CRC) is the third most common cancer worldwide, with over 1.9 million new cases diagnosed and 935,000 deaths in 2020 according to the World Cancer Research Fund. Cellular senescence has also been associated with colorectal carcinogenesis [13,14]. It has been reported that TIS increases CRC cell invasion *in vitro*, and elevated expression of senescence markers is associated with bad prognosis in this type of cancer [9,14]. Moreover, 5-fluorouracil (5-FU), commonly used in CRC treatment, can induce senescence, potentially contributing to 5-FU resistance and tumor recurrence [9]. According to Tato-Costa et al., the SASP elicited by 5-FU treatment may drive EMT, a process they observed not only *in vitro* but also in tissue samples from rectal cancer patients [9]. Senotherapy—therapeutic strategies that target senescent cells—has therefore attracted considerable attention. Selective elimination of senescent cells (senolytics) or attenuation of the SnC secretome (senomorphics) is considered a promising approach to reduce tumor progression.

**Figure 1:**
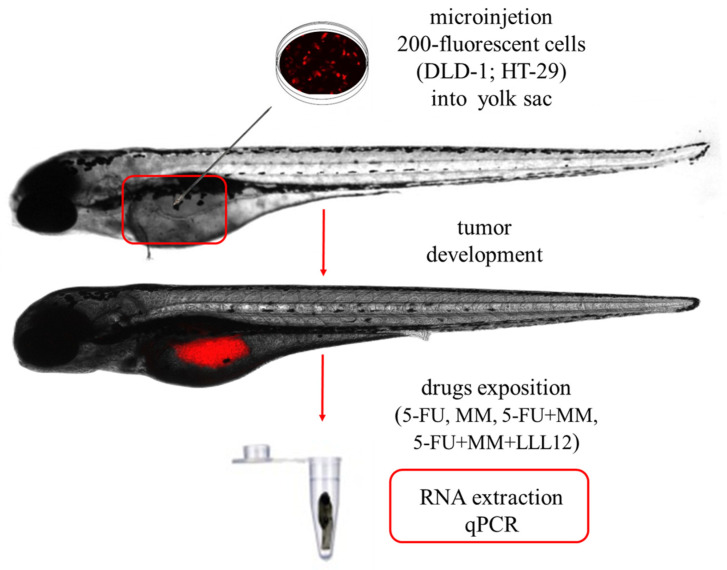
The schematic protocol of zebrafish study. Abbreviations: 5-FU—5-fluorouracil; DLD-1/HT-29—colorectal cell lines; LLL12—STAT-3 inhibitor, MM—MM-129 (pyrazolo[4,3-e]tetrazolo[4,5-b][1,2,4]triazine sulfonamide).

MM-129, structurally defined as a pyrazolo[4,3-e]tetrazolo[4,5-b][1,2,4]triazine-based sulfonamide, represents a novel compound synthesized as a potential anticancer agent and showing promising properties in preclinical studies. It demonstrated a good safety profile in zebrafish and mouse toxicity assays [15]. We have recently reported that MM-129 effectively inhibits colon cancer development via attenuation of intracellular pathways promoting tumorigenesis, such as PI3K/AKT/mTOR (Figure 1) [16]. Since these molecules play a significant role in the promotion of cellular senescence and SASP factor production, we decided to evaluate senotherapeutic properties of MM-129.

In the present study, we assessed this 1,2,4-triazine compound as a senotherapeutic candidate for the treatment of colon cancer. MM-129 effectively prevented cellular senescence and suppressed the SASP through a SIRT1/STAT3-dependent mechanism.

## 2. Materials and Methods

### 2.1. Cell Culture

Both DLD-1 (ATCC, Cat# CCL-221, RRID:CVCL_0248) and HT-29 (ATCC, Cat# HTB-38, RRID:CVCL_0320) human colorectal adenocarcinoma cell lines were obtained from the American Type Culture Collection (ATCC, Manassas, VA, USA). The characteristics of these cell lines have been described previously [16,17]. For all experiments, both cell lines were used at passages 6–8 after thawing and were handled under sterile conditions. No further authentication or mycoplasma testing was performed during the course of the study, which we acknowledge as a limitation. DLD-1 cells were cultured in RPMI 1640 medium (Sigma, St. Louis, MO, USA) and HT-29 cells in McCoy’s 5a medium (ATCC). Both media were supplemented with 10% fetal bovine serum (FBS; Gibco, Thermo Fisher Scientific, Waltham, MA, USA) and 1% antibiotics (penicillin/streptomycin; Sigma-Aldrich, St. Louis, MO, USA). Cells were cultured in 100 mm plates (Sarstedt, Newton, NC, USA) and maintained at 37 °C in 5% CO_2_ and 90–95% humidity. Experiments were initiated at ≤ 70% confluency to avoid senescence due to over-confluence. Cells were treated with 5-FU (1 µM; Sigma-Aldrich, MilliporeSigma, St. Louis, MO, USA), MM-129 (10 µM; synthesized as previously described [16]), or both for 6 days, then analyzed for senescence [9,18,19,20]. Media were replaced every 48 h.

### 2.2. Detection of β-Galactosidase Activity as a Marker of Cellular Senescence

Senescence-associated β-gal activity, indicative of enhanced lysosomal mass, was analyzed. SA-β-gal staining was carried out using the Senescence Cell Histochemical kit (CS0030; Merck KGaA, Darmstadt, Germany), following the supplier’s instructions. Cells were fixed with 4% paraformaldehyde (10 min, RT) after medium removal, and subsequently rinsed three times with PBS. After fixation, cells were incubated overnight at 37 °C (in the absence of CO_2_) with a freshly prepared SA-β-gal staining solution (pH 6.0) containing 1 mg/mL X-Gal (5-bromo-4-chloro-3-indolyl-β-D-galactoside, dissolved in dimethylformamide), 40 mM citric acid/sodium phosphate buffer, 5 mM potassium ferrocyanide, 5 mM potassium ferricyanide, 150 mM NaCl, and 2 mM MgCl_2_. Following staining, cells were rinsed twice with PBS (pH 6.0; Gibco, Thermo Fisher Scientific, Waltham, MA, USA). The proportion of senescent cells was then determined by counting blue-green-stained cells using an inverted phase-contrast microscope (Leica DMIL LED; Leica Biosystems, Wetzlar, Germany) at 4× magnification. The percentage of SA-β-gal-positive cells was calculated by counting 1000 cells in seven randomly selected fields.

### 2.3. Capillary Western Blot

The cells were lysed in 100 µL RIPA buffer enriched with protease and phosphatase inhibitors (Sigma-Aldrich, St. Louis, MO, USA). Cellular membranes were disrupted by sonication. The total protein concentration was determined using the bicinchoninic acid (BCA) method according to the manufacturer’s protocol (Thermo Fisher Scientific, Waltham, MA, USA). Samples were equalized to 0.4 mg/mL total protein and loaded into a cartridge [21]. Protein samples were separated by capillary electrophoresis using the 12–230 kDa Jess Separation Module (ProteinSimple, Bio-Techne, San Jose, CA, USA) following the manufacturer’s instructions. Target proteins were detected with the following antibodies: mouse anti-Cdh1 (Abcam, Cambridge, UK; Cat# ab76055), rabbit anti-phospho-Sirt1 (Merck, Darmstadt, Germany; Cat# SAB5701821), rabbit anti-phospho-Stat3 (Invitrogen, Carlsbad, CA, USA; Cat# 710093) and monoclonal antibody against beta-actin (Sigma-Aldrich, St. Louis, MO, USA; Cat# A2228). For detection, the anti-rabbit module for the Jess (DM-001, DM-002, ProteinSimple) kit was used, which includes luminol-S, peroxide, antibody diluent 2, streptavidin-HRP, and anti-rabbit or anti-mouse secondary antibody. For analysis, the chemiluminescence of secondary antibodies signal peaks were chosen, and in the next step, they were converted into bands as observed in a Western Blot analysis. To confirm loaded protein level and to verify normalized protein amount, detection of housekeeping protein β-actin was performed. Results were analyzed using Compass for SW software v5.0.1. The values were obtained from three independent experiments done in duplicate (*n* = 6). This technology was chosen because it offers highly sensitive, quantitative, and automated protein detection; minimizes manual handling and variability; and enables accurate measurement from low sample inputs—features well-documented and recommended for translational research.

### 2.4. RNA Extraction and Quantitative Analysis

Colon cancer DLD-1 and HT-29 cell lines were labeled with Vybrant Dil (Invitrogen). Vybrant Dil staining was carried out according to the supplier’s protocol, and the cells were adjusted to a concentration of 1 × 10^6^/mL in culture medium. At 48 hpf, zebrafish embryos were manually dechorionated for xenotransplantation. Cancer cells were then loaded into glass injection needles produced with a P-1000 puller (Sutter Instrument Company, Novato, CA, USA).

After injection, the zebrafish larvae with transplanted cells were randomly assigned to control and drug treatment groups (5-FU, MM, 5-FU + MM, 5-FU + MM + LLL12). The zebrafish with the xenografts were maintained in an E3 buffer at 34 °C until the end of the experiments.

Total RNA from 20 fish with xenograft in each studied group was isolated using ExtractMe Total RNA kit (Blirt S.A., Gdańsk, Poland), according to the manufacturer’s protocol. The isolated RNA was used for cDNA synthesis through High-Capacity cDNA Reverse Transcription Kit with the addition of an RNase Inhibitor (Applied Biosystems, Thermo Fisher Scientific, Waltham, MA, USA) [21]. The human GAPDH was used for normalization of p21, SIRT1, STAT3, CDH1, TNF-α and IL-6 gene analysis (the primer sequences are listed in Table 1).

All primers used for qPCR were tested for specificity and sensitivity. Quantitative real-time PCR (qPCR) expression analysis was then performed with the LightCycler^®^ 480 II instrument (Roche, Basel, Switzerland) in triplicates on 96-well plates using PowerUp SYBR Green Master Mix (Applied Biosystems). Relative mRNA expression was calculated using delta CT subtraction and normalized [21].

### 2.5. Cytometry Analysis

The CellEvent Senescence Green Flow Cytometry Assay Kit (Invitrogen, Cat #C1084) enables flow cytometric detection of cellular senescence via β-galactosidase hydrolysis. The assay was performed in accordance with the manufacturer’s instruction. Cells were trypsinized and counted by Scepter 3.0 handheld automated cell counter (Millipore, Burlington, MA, USA), then 4 × 10^4^ cells were washed and suspended in 100 μL 1% BSA in PBS. The prepared cells were fixed with 3% paraformaldehyde in PBS for 10 min at room temperature in the dark. Fixative solution was removed by washing cells in 2 mL of 1% BSA in PBS, then cells were probed with 1.0 μM CellEvent Senescence Green fluorescent substrate, as described by the manufacturer, and assessed by flow cytometry using a 488 nm laser and 530/30 nm filter. Percentages of SA-β-gal + /SA-β-gal-cells were quantified according to the median fluorescence intensity (MFI) of CellEvent Senescence Green staining in probed cells compared to negative control, unstained non-senescent cells. Flow cytometry analysis was performed on a BD FACSCanto II using FACSDiva software version 6.1.3 (both BD Biosciences Systems, San Jose, CA, USA) and FCS Express 7 software, version 7.10.0007 (De Novo Software, Pasadena, CA, USA). Equipment calibration was performed using the BD Cytometer Setup and Tracking Beads (BD Biosciences, San Diego, CA, USA).

### 2.6. Immunohistochemistry

**Figure 2:**
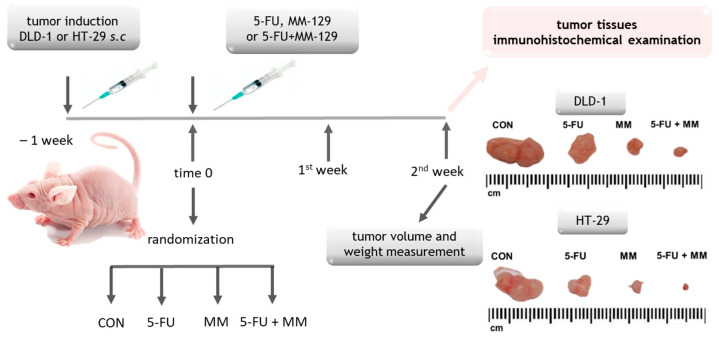
Schematic representation of *in vivo* experiment from which tissues were obtained for further analysis. Abbreviations: 5-FU—5-fluorouracil; DLD-1/HT-29—colorectal cell lines; MM—MM-129 (pyrazolo[4,3-e]tetrazolo[4,5-b][1,2,4]triazine sulfonamide).

Tumor samples were generated in a previously described xenograft model (Figure 2) [16]. In short, Cby.Cg-Foxn1nu/cmdb mice received subcutaneous injections on the dorsal flank with 50 μL of PBS suspensions containing either DLD-1 or HT-29 cells, following the procedure of Shinohara et al. (2012) [22]. For each cell line, the animals were randomly assigned to four treatment arms (*n* = 10 per group). Drug administration was initiated once tumors reached approximately 5 mm in diameter, a threshold commonly accepted for therapeutic testing. Mice were then treated intraperitoneally with 5-FU, MM-129, or their combination for two weeks. At the study endpoint, euthanasia was performed via intraperitoneal overdose of pentobarbital. All animal experiments adhered to institutional guidelines and were approved by the Local Ethical Committee on Animal Testing (Permit No. 92/2019).

During necropsy, excised tumor samples were placed in 10% neutral-buffered formalin to preserve tissue architecture for immunohistochemistry. After fixation, the material was processed into paraffin blocks, and thin sections (4 μm) were prepared on silanized glass slides. The slides were dried overnight at 60 °C and then subjected to standard deparaffinization and rehydration steps. Endogenous peroxidase activity was quenched with 3% hydrogen peroxide, and non-specific binding was minimized by pre-incubation with 1% bovine serum. For antigen detection, sections were incubated for 30 min at room temperature with a rabbit polyclonal anti-β-galactosidase antibody (Biorbyt, Cambridge, UK; Cat# orb556162; dilution 1:200). The detection system used was a polymer technique (ImmPRESS^®^ HRP Goat Anti-Rabbit IgG Polymer Detection Kit; Vector Laboratories, Newark, CA, USA). The color reaction for peroxidase was performed with DAB chromogen (ImmPACT^®^ DAB Substrate, Peroxidase (HRP), VectorLaboratories, Newark, CA, USA). The cell nuclei were counterstained with hematoxylin. The intensity of SA-β-gal staining was assessed semi-quantitatively by two independent observers using a 4-tier visual scoring system commonly applied in histopathology: 0 (no staining), 1+ (weak; <10% cells), 2+ (moderate; 10–50% cells), and 3+ (strong; >50% cells). Results were categorized accordingly as weak (1+), moderate (2+), or strong (3+). Any discrepancies were resolved by consensus, and representative images were selected for illustration.

### 2.7. Statistical Analysis

Statistical analyses were carried out using GraphPad Prism software (version 10.2.2; GraphPad Software, San Diego, CA, USA). The distribution of data was verified with the Shapiro–Wilk test. For variables showing normality, comparisons were made with one-way ANOVA, and results are expressed as mean ± standard deviation (SD). Tukey’s post hoc procedure was applied to evaluate group differences, with statistical significance defined as *p* <  0.05.

## 3. Results

### 3.1. MM-129 Counteracts 5-FU-Initiated Senescence of Colon Cancer Cells

**Figure 3:**
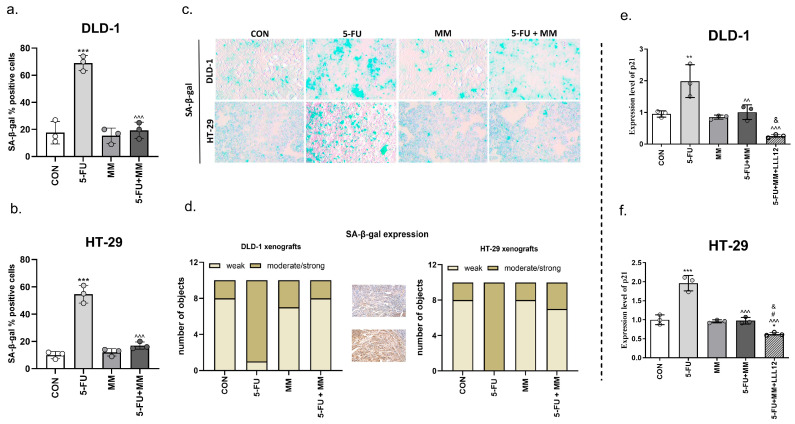
Impact of 5-FU, MM-129 (MM), combination of these compounds (5-FU + MM) and LLL12-STAT3 inhibitor (5-FU + MM + LLL12) on cellular senescence markers in DLD-1 and HT-29 colon cancer cells. The cells were stained with the CellEvent Senescence Green probe. A fluorescence signal from the enzyme-cleaved product was analyzed using a confocal system. Representative images of the percentage of DLD-1 and HT-29 positive for SA-β-gal; (**c**) and bar graphs (**a**,**b**). Senescence was quantitated by visual inspection using an inverted phase-contrast microscope with 4× magnification. Immunohistochemical staining of SA-β-gal in solid tumor tissues obtained from DLD-1 and HT-29 xenografts treated with 5-FU, MM-129 and combination of these compounds. Protein expression was assessed with an Olympus BX41 light microscope at 100 × magnification and was classified as negative when the expression was negative, or positive in < 10% of cells, and positive when the reaction was positive in ≥10% of cells (**d**). p21 level in DLD-1 (**e**) and HT-29 (**f**) zebrafish xenografts after incubation with 5-FU, MM-129 (MM) their combination (5-FU + MM and 5-FU + MM + LLL12). The results are presented as means ± SDs, *n* = 6. * *p* < 0.05, ** *p* < 0.01, *** *p* < 0.001 vs. CON; ^^^^ *p* < 0.01, ^^^^^ *p* < 0.001 vs. 5-FU, ^#^ *p* < 0.05 vs. MM-129, ^&^ *p* < 0.05 vs. 5-FU + MM-129. Abbreviations: CON—control; MM—MM-129; 5-FU—5-fluorouracil; LLL12—STAT3 inhibitor.

Senescent cells typically exhibit increased lysosomal β-galactosidase activity, detectable as SA-β-gal. Following treatment with 5-FU, both DLD-1 and HT-29 cultures showed a significant rise in SA-β-gal-stained cells relative to the control group (*** *p* < 0.001 vs. CON). MM-129 reversed the effect induced by 5-FU and dramatically decreased the proportion of SnCs in DLD-1 and HT-29 from 69% to 19% (^^^^^ *p* < 0.001 vs. 5-FU) and from 55% to 17% (^^^^^ *p* < 0.001 vs. 5-FU), respectively (Figure 3a–c). Compared to the control group, 5-FU-treated cells exhibited blue staining and appeared larger and flatter (Figure 3c). Analysis of SA-β-gal expression in solid tumor tissues revealed moderate/strong expression for SA-β-gal in 90% of DLD-1 xenografts (9/10) and in all HT-29 xenografts (10/10) treated with 5-FU. MM-129 alone did not affect the expression level of this marker compared to control. However, it abolished the effect of 5-FU in the group treated with 5-FU + MM-129 (2/10 in DLD-1 xenografts and 3/10 in HT-29 xenografts) (Figure 3d).

p21 is a well-known regulator of cell cycle progression and a central mediator of cellular senescence. It exerts its function by binding to and inhibiting cyclin-CDK2, cyclin-CDK1, and cyclin-CDK4,6 complexes [23]. 5-FU treatment significantly enhanced p21 level in DLD-1 and HT-29 zebrafish xenografts compared with control (** *p* < 0.01, *** *p* < 0.001 vs. CON in DLD-1 and HT-29, respectively). In the groups of cells exposed to 5-FU + MM-129 the abolition of the effect induced by 5-FU alone was observed (^^^^ *p* < 0.01 vs. 5-FU for DLD-1; ^^^^^ *p* < 0.001 vs. 5-FU for HT-29). Inhibition of STAT3 using the small-molecule inhibitor LLL12 (5µM) significantly decreased p21 level, compared with control (* *p* < 0.05 vs. CON, HT-29), 5-FU (^^^^^ *p* < 0.001 vs. 5-FU; DLD-1 and HT-29), MM-129 (^#^ *p* < 0.05 vs. MM-129; HT-29) and 5-FU + MM-129 (^&^ *p* < 0.05 vs. 5-FU + MM-129, both in DLD-1 and HT-29).

### 3.2. MM-129 Reduces the Number of SA-β-Gal-Positive Senescent Colon Cancer Cells Induced by 5-FU

**Figure 4:**
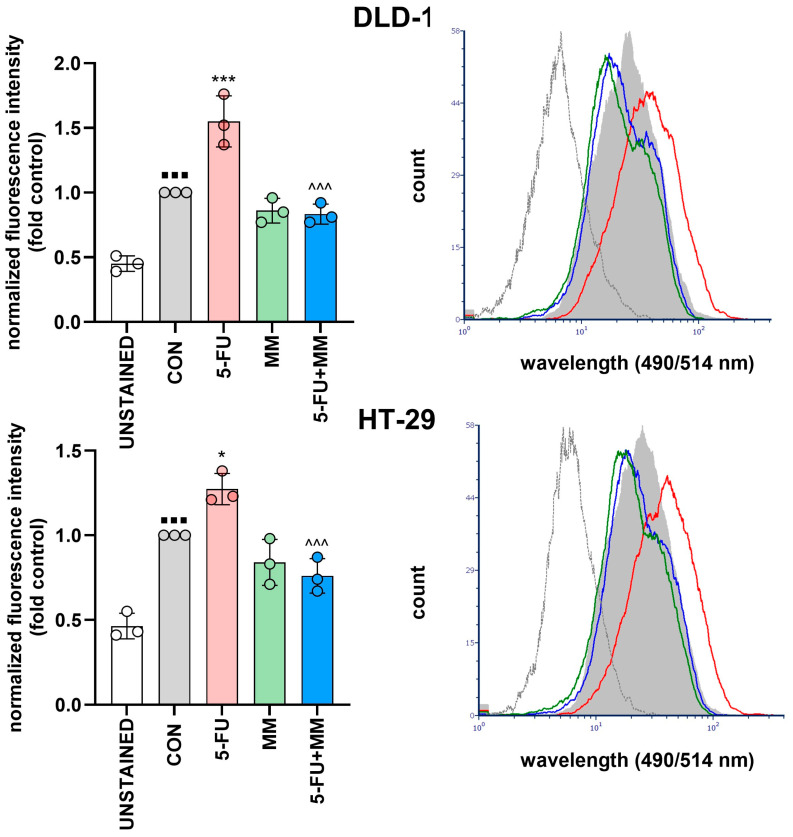
Representative histograms depict β-galactosidase activity in DLD-1 and HT-29 cells after treatment with 5-FU, MM-129, or their combination. Unstained population (UNSTAINED) is shown in white, control in grey, whereas treated groups are marked in red (5-FU), green (MM), and blue (5-FU + MM). Differences in mean fluorescence intensity (MFI) reflect the level of enzymatic hydrolysis. Values represent the mean of three independent experiments (triplicate measurements). * *p* < 0.05, *** *p* < 0.001 vs. CON; ^^^^^ *p* < 0.001 vs. 5-FU, ^▪▪▪^ *p* < 0.001 vs. UNSTAINED. Abbreviations: UNSTAINED—unstained negative control; CON—control; MM—MM-129; 5-FU—5-fluorouracil; MFI—mean fluorescence intensity.

Next, we applied flow cytometry-based senescence assay to detect and quantify senescence in DLD-1 and HT-29 cells after exposure to 5-FU, MM-129 and combination of these drugs. The percentage of cells expressing SA-β-gal significantly increased in cells treated with 5-FU compared to control in both DLD-1 and HT-29 (*** *p* < 0.001, * *p* < 0.05 vs. CON, respectively). In both lines, clear decrease in the number of SA-β-gal-positive cells after adding MM-129 to 5-FU was observed (^^^^^ *p* < 0.001 vs. 5-FU in DLD-1 and HT-29). Senescence-associated β-galactosidase expression following MM-129 treatment was comparable to that observed in the control group (Figure 4).

### 3.3. MM-129 Inhibits the Senescence-Associated Secretory Phenotype Induced by 5-FU

**Figure 5:**
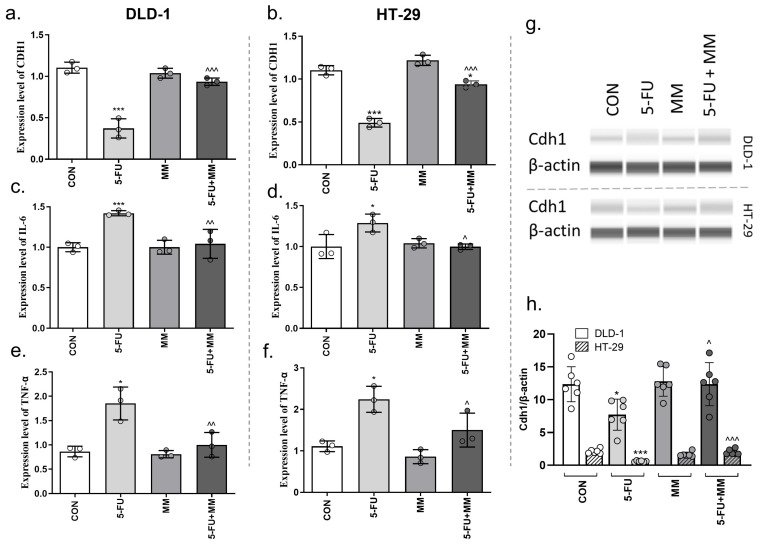
CDH1 (**a**,**b**), IL-6 (**c**,**d**) and TNF-α mRNA (**e**,**f**) levels in DLD-1 and HT-29 cells after treatment with 5-FU, MM-129 (MM), and their combination (5-FU + MM). The results are presented as means ± SDs, *n* = 6. Cdh1 and β-actin expression as determined by Western Blot (**g**,**h**). The values were obtained from three independent experiments performed in duplicate. Images and quantification were obtained by capillary protein separation and immunodetection. * *p* < 0.05; *** *p* < 0.001 vs. CON; ^^^ *p* < 0.05; ^^^^ *p* < 0.01; ^^^^^ *p* < 0.001 vs. 5-FU. Abbreviations: CON—control; MM—MM-129; 5-FU—5-fluorouracil.

SASP is a crucial pathological feature of SnCs. Despite the arrest of cell cycle and proliferation, SnCs can promote tumor development mainly via a set of factors that affect both themselves and their surrounding environment. SASP can induce EMT, including via downregulation of E-cadherin (Cdh1). The incubation of DLD-1 and HT-29 zebrafish xenografts with 5-FU downregulated CDH1 mRNA expression compared with the control (*** *p* < 0.001 vs. CON; Figure 5a,b). qPCR analysis showed that a simultaneous use of 5-FU + MM-129 increase CDH1 mRNA expression compared with 5-FU alone in both animal groups (^^^^^ *p* < 0.001 vs. 5-FU). Using Western Blot, we found a decrease in Cdh1 expression after 5-FU stimulation compared with the control (* *p* < 0.05, *** *p* < 0.001 vs. CON in DLD-1 or HT-29, respectively; Figure 5g,h). 5-FU + MM-129 caused reduction in 5-FU effect and elevation of the Cdh1 level compared with 5-FU (^^^ *p* < 0.05 vs. 5-FU in DLD-1, ^^^^^ *p* < 0.001 vs. 5-FU in HT-29).

IL-6 is the most prominent cytokine of the SASP. Through this pleiotropic proinflammatory cytokine, SnCs can directly affect neighboring cells that express the IL-6R (gp80) and gp130 signaling complex at their surface [24]. Moreover, IL-6 activates EMT processes, promoting the acquisition of mesenchymal features in cancer cells [25]. To investigate whether MM-129 is able to inhibit SASP, the mRNA level of this cytokine was determined by qPCR (Figure 5c,d). 5-FU induced a significant increase in IL-6 mRNA level compared with control (*** *p* < 0.001; * *p* < 0.05 vs. CON in DLD-1 and HT-29 xenografts, respectively), while adding MM-129 reversed the increase in this factor (^^^^ *p* < 0.01 vs. 5-FU in DLD-1; ^^^ *p* < 0.05 vs. 5-FU in HT-29).

TNF-α, another SASP component, acts as a pro-inflammatory cytokine that can drive production of other mediators such as IL-6 and IL-1β [26]. In both DLD-1 and HT-29 zebrafish xenografts 5-FU significantly enhanced TNF-α mRNA versus control (* *p* < 0.001 vs. CON in DLD-1 and HT-29). Co-treatment with MM-129 counteracted this effect (^^^^ *p* < 0.01 vs. 5-FU in DLD-1; ^^^ *p* < 0.05 vs. 5-FU in HT-29).

### 3.4. MM-129 Induces Senotherapeutic Effect via SIRT1/STAT3 Inhibition

**Figure 6:**
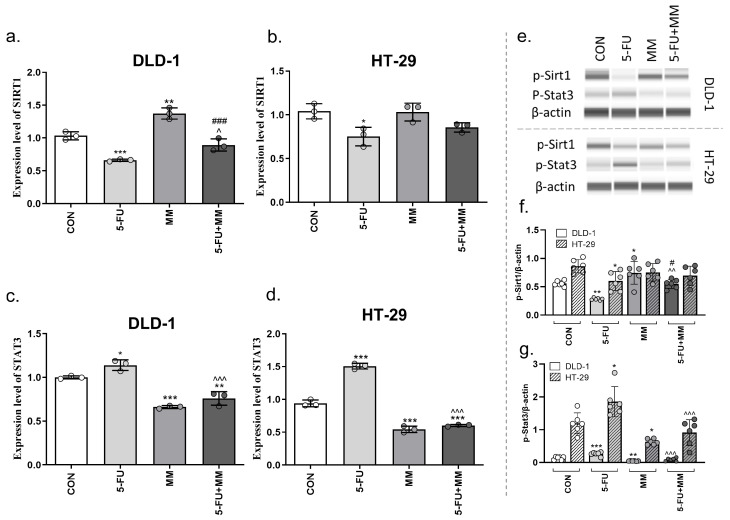
SIRT1 (**a**,**b**) and STAT3 (**c**,**d**) mRNA levels in DLD-1 and HT-29 cells after incubation with 5-FU, MM-129 (MM) and their combination (5-FU + MM). The results are presented as means ± SDs, *n* = 6. p-Sirt1, p-Stat3 and β-actin expression as determined by Western Blot (**e**–**g**). The values were obtained from three independent experiments performed in duplicate. Images and quantification were obtained by capillary protein separation and immunodetection. * *p* < 0.05; ** *p* < 0.01, *** *p* < 0.001 vs. CON; ^^^ *p* < 0.05; ^^^^ *p* < 0.01; ^^^^^ *p* < 0.001 vs. 5-FU, ^#^ *p* < 0.05; ^###^ *p* < 0.001 vs. MM-129. Abbreviations: CON—control; MM—MM-129; 5-FU—5-fluorouracil.

We also evaluated the status of the SIRT1 and STAT3 signaling pathways to determine the mechanism by which MM-129 inhibits 5-FU-induced senescence (Figure 6). DLD-1 and HT-29 xenografts exposed to 5-FU exhibited downregulation of SIRT1 mRNA level (*** *p* < 0.001; * *p* < 0.05 vs. CON in DLD-1 or HT-29, respectively). In turn, MM-129 alone (** *p* < 0.01 vs. CON) and in combination with 5-FU significantly upregulated SIRT1 mRNA level in DLD-1 xenografts (^^^ *p* < 0.05 vs. 5-FU; ^###^ *p* < 0.001 vs. MM-129). A similar trend was observed in HT-29, but these were not statistically significant changes. These results were confirmed using Western Blot analysis. p-Sirt1 expression was clearly reduced by 5-FU compared with control cells in both lines (** *p* < 0.01; * *p* < 0.05 vs. CON in DLD-1 or HT-29, respectively). MM-129 alone increases the expression level of p-Sirt1 only in DLD-1 (* *p* < 0.05 vs. CON). Simultaneous treatment with 5-FU and MM-129 resulted in the elimination of the effect induced by 5-FU in DLD-1 cells (^^^^ *p* < 0.01 vs. 5-FU; ^#^ *p* < 0.05 vs. MM-129) but not in HT-29.

With 5-FU added to DLD-1 and HT-29 xenografts, STAT3 mRNA levels were significantly increased (* *p* < 0.05 vs. CON in DLD-1 and *** *p* < 0.001 vs. CON in HT-29). The opposite effect was observed in the groups of animals exposed to MM-129, where the expression of STAT3 was significantly lower than in both DLD-1 and HT-29 control xenografts (*** *p* < 0.001 vs. CON). In groups treated with 5-FU + MM-129, STAT3 mRNA level decreased in comparison with control (** *p* < 0.01; *** *p* < 0.001 vs. CON in DLD-1 and HT-29, respectively), and abolishment of the effect produced by 5-FU was reported (^^^^^ *p* < 0.001 vs. 5-FU). Moreover, we found that STAT3 mRNA expression level was in line with its protein expression. 5-FU-treated groups, both DLD-1 and HT-29, showed increased p-Stat3 expression (*** *p* < 0.001, * *p* < 0.05 vs. CON in DLD-1 or HT-29, respectively); MM-129 alone decreased the level of this protein (** *p* < 0.01 vs. CON in DLD-1, * *p* < 0.05 vs. CON in HT-29), while MM-129 in combination with 5-FU counteracted the effect generated by 5-FU (^^^^^ *p* < 0.001 vs. 5-FU in both lines).

## 4. Discussion

Therapies that target senescence have been shown to extend lifespan and reduce tissue injury in various animal models [27,28]. Early clinical trials also confirm that senotherapeutic approaches could be beneficial in many human diseases, including cancer [29,30]. In the context of tumors, senotherapeutic strategies, known as one-two-punch cancer therapy, typically involve a combination of a senescence-inducing drug and another drug targeting SnCs [31]. Cancer therapies at clinical doses, while effectively inducing tumor cell killing (first punch), can also lead to senescence in both tumor and normal tissues [32]. Pathologic accumulation of SnCs can promote tumorigenesis and cancer recurrence [33,34]. Moreover, the burden of SnCs can also worsen the toxic side effects of treatments, increase therapy resistance, and is associated with a poor prognosis [35]. Selective clearance of SnCs with a senotherapeutic agent (second punch) is crucial for reducing the risk of tumor progression. This strategy can help prevent tumor relapse, metastasis, and development of resistance to treatment. It has been shown that the elimination of SnCs suppresses lung metastasis of melanoma cells, attenuates glioblastoma recurrence, and increases survival in mice bearing glioblastoma multiforme tumors [34,36,37]. Moreover, selective clearance of SnCs from normal tissues may help prevent therapy-induced side effects, alleviate existing damage, and support the restoration of tissue homeostasis [37,38,39].

Tato-Costa J. et al. reported that low doses of 5-FU can induce cellular senescence in colorectal cancer cells and, in an SASP-dependent manner, lead to paracrine induction of EMT and increased cell invasiveness *in vitro* [9]. Furthermore, other authors have reported that 5-FU could contribute to the senescence of human endothelial cells, which may subsequently lead to chemotherapy-related cardiovascular disease [18]. In our study, we demonstrated that the novel 1,2,4-triazine derivative MM-129 reduced 5-FU–induced senescence-associated features, significantly lowering the proportion of SA-β-gal–positive cells in both DLD-1 and HT-29 cells (Figure 3a–c). Using flow cytometry, we observed that MM-129, in contrast to 5-FU, significantly decreased the number of SA-β-gal-positive cells, whereas administration of 5-FU together with MM-129 reversed the effect evoked by 5-FU alone (Figure 4). Moreover, immunohistochemical examination showed that MM-129 alone had no effect on the expression of SA-β-gal compared to the control in solid tumor tissues obtained from DLD-1 and HT-29 xenografts. The strongest expression of this marker was found in samples obtained from mice exposed to 5-FU. Addition of MM-129 to 5-FU resulted in a significant decrease in the level of SA-β-gal expression in tissues obtained from both DLD-1 and HT-29 xenografts (Figure 3d).

The senescent phenotype is characterized by a permanent cell cycle arrest and elevated levels of tumor suppressors, including p21 [40]. We observed significant increase in p21 level after exposure to 5-FU. Simultaneous exposure to 5-FU + MM-129 or 5-FU + MM + LLL12 counteracted the response induced by 5-FU alone in both DLD-1 and HT-29 zebrafish xenografts (Figure 3e,f). Our findings are consistent with both the ‘one-two punch’ concept and findings from other researchers. Xia et al. reported that 5-FU led to an increase in SA-β-gal-positive cells, elevated expression of senescence-associated markers, including proteins (p16), aging-related genes (p53, p21), and SASP factors (IL-1β, IL-6, TNF-α),while atorvastatin calcium (Ator) reversed the increase in these factors [19]. In a breast cancer study, the senolytic agent navitoclax cleared doxorubicin-induced senescent cells (SnCs) in mice bearing MMTV-PyMT breast carcinomas. This intervention significantly delayed cancer metastasis and recurrence in a preclinical mouse model [41]. Dasatinib monotherapy was effective in triple-negative breast cancer (TNBC) preclinical studies. It restored paclitaxel sensitivity in TNBC-resistant cell lineages and reduced the number of stem cells [42].

**Figure 7:**
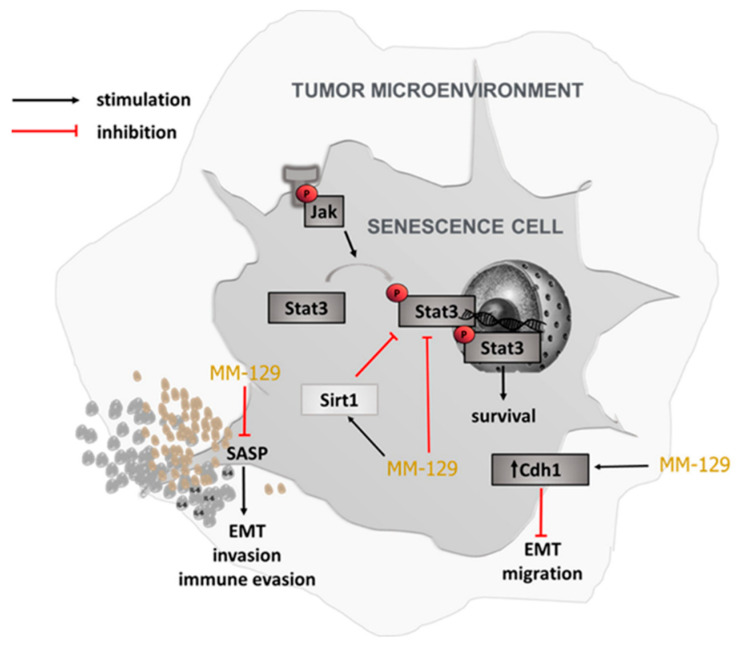
Schematic representation of the potential senotherapeutic actions of MM-129. Abbreviations: Cdh1—E-cadherin, a type I transmembrane protein involved in epithelial adhesion; EMT—epithelial–mesenchymal transition, a process linked to tumor progression; JAK—Janus kinases, intracellular signaling mediators; SASP—senescence-associated secretory phenotype, comprising pro-inflammatory factors secreted by senescent cells; Sirt1—a member of the sirtuin family with roles in metabolism and stress resistance; Stat3—signal transducer and activator of transcription 3, a transcription factor downstream of multiple cytokine pathways.

SnCs may contribute to oncogenesis primarily through the SASP, which creates an immunosuppressive environment. The key SASP factors, IL-6 and TNF-α, can amplify cellular senescence in SASP-producing cells via an autocrine mechanism. At the same time, they promote proliferation in neighboring cells through paracrine signaling [26,43]. This dual role of IL-6 has also been described by Kuilman et al., who reported that IL-6 promotes senescence through an autocrine loop and enhances cell proliferation via paracrine signaling in a melanoma cell model [44]. In the pituitary, IL-6 produced by folliculostellate cells acts via paracrine fashion to trigger proliferation in pituitary tumor cell models [45]. IL-6 also plays a crucial role in pro-metastastic and EMT. Knockout of IL-6 significantly attenuated the pro-metastatic effect of sorafenib and increased the susceptibility of liver cancer cells to it [46]. TNF-α released by senescent cells, activates nuclear factor-kappa B (NF-κB) and mitogen-activated protein kinase (MAPK) signaling pathways in local environment [26]. This chronic TNF-α signaling creates a proinflammatory microenvironment that affects neighboring cells, alters immune surveillance reducing the clearance of senescent and premalignant cells and supports tumor initiation progression and metastasis. TNF-α together with other SASP cytokines promote EMT and tissue remodeling [47,48]. Furthermore, blocking TNF-α with adalimumab led to a marked decrease in IL-6 secretion in long-term HUVEC cultures [23]. In turn, administration of infliximab decreased breast cancer metastasis to lungs in mice by ~60% [49]. In our study, 5-FU robustly induced cellular senescence and SASP, as evidenced by increased SA-β-gal activity and elevated IL-6 and TNF-α expression. By contrast, MM-129 significantly decreased IL-6 and TNF-α expression and counteracted the effect produced by 5-FU. Similar results were observed regarding E-cadherin (Cdh1), a key marker involved in the regulation of EMT (Figure 7).

EMT represents a fundamental step in tumor progression, as epithelial cells lose their polarity and adopt mesenchymal traits, characterized by greater motility and invasive potential. Among the key molecules, E-cadherin—a type I transmembrane protein—plays a central role in preserving intercellular adhesion and epithelial architecture. Reduced expression of this classical epithelial marker is a hallmark of EMT and is associated with increased tumor invasiveness. E-cadherin has been identified as a tumor suppressor gene affecting EMT, and is closely related to TNM (Tumor, Node, Metastasis) stage, lymph node metastasis, extracapsular invasion and low disease-free survival rate [50]. Using both Western Blot and RT-PCR techniques, we found that colon cancer cells and zebrafish xenograft samples treated with 5-FU exhibited decreased E-cadherin expression at both the mRNA and protein levels. The addition of MM-129 to 5-FU resulted in a significant increase in E-cadherin expression compared to treatment with 5-FU alone (Figure 5a,b,g,h). These observations are consistent with the findings of Christou et al., who reported that E-cadherin expression decreased following 5-FU treatment in CRC cell lines derived from advanced tumor stages [51]. Restoration of E-cadherin represents a potential therapeutic approach in colon cancer. Numerous compounds have been shown to exert anti-tumor activity by increasing E-cadherin levels, including everolimus (renal cancer), galeterone (prostate cancer), and resveratrol (ovarian cancer), among others [52]. Used in our study, the 1,2,4-triazine derivative displayed senotherapeutic activity by regulating key molecules involved in signaling pathways, including E-cadherin.

mTOR is an important regulator of the SASP, and mTOR inhibitors specifically eliminate senescent cancer cells [28]. Rapamycin (mTOR inhibitor) was reported to reduce SASP both *in vitro* and in mice following the replicative senescence [28,53,54]. The mTORC1 phosphorylation cascade integrates signals from the persistent DNA damage response and promotes the increase in mitochondrial mass, which in turn, contributes to cellular senescence and the secretion of proinflammatory IL-6, IL-8 and MCP-1. Fung et al. have observed that both concurrent and sequential therapy with chemotherapy (docetaxel) and the mTOR inhibitor temsirolimus resulted in strong antitumor effects in prostate and breast cancer cells and xenografts [55]. Atorvastatin calcium, also in an mTOR-dependent manner, inhibited senescence in human intestinal epithelial cells and the intestinal epithelium of mice. Since we have previously reported that MM-129 exerts the ability to inhibit the mTOR signaling cascade, we hypothesize that this is one of the mechanisms responsible for its senotherapeutic effect [16].

In this study, we showed that the SIRT1/STAT3 signaling pathway is also involved in MM-129’s ability to eliminate SnCs. We found that SIRT1 was downregulated in colon cancer cells exposed to 5-FU. In turn, the addition of MM-129 counteracted this effect and clearly increased SIRT1 levels compared to the 5-FU-treated group. SIRT1, a member of the mammalian sirtuin family, is an NAD+-dependent deacetylase that plays key roles in aging-related diseases and cellular senescence [56].

It has been reported that SIRT1 expression is decreased, whereas SASP factor expression is increased, during cellular senescence. Moreover, IL-6 and IL-8 rapidly accumulated in SIRT1-depleted cells both in mRNA and protein levels, exceeding the level in control cells, indicating that SIRT1 negatively regulates the expression of SASP components at the transcriptional level [57]. Using the Western Blot technique herein, we noticed that 5-FU significantly decreased the expression of SIRT1. This finding is in line with Altieri et al., who showed that 5-FU affected SIRT-1. They reported that 5-FU-elicited senescence occurred via activation of p38 and JNK and was associated with decreased levels of eNOS and SIRT1 [18].

Previous reports have documented the crosstalk between SIRT1 and signal transducer and activator of transcription 3 (STAT3) in various types of cancer. SIRT1 has been shown to inhibit the activation of STAT3 to repress the gastric cancer growth [58]. Ray et al. demonstrated that, in mouse liver cells, inhibition of SIRT1 activity using antisense oligomers or pharmacological inhibitors increased STAT3 acetylation and phosphorylation [59]. The roles of STAT family members (and related molecules) have also been shown in premature senescence [60]. The constitutive activation of STAT3 can upregulate the expression of anti-apoptotic proteins and cyclins thereby regulating the cell cycle, promoting cell proliferation, and inhibiting apoptosis. In addition to cancer cells, STAT3 expression in CAFSs (senescent cancer-associated fibroblasts) can enhance the viability of cancer cells, which is related with the senescence-associated secretory phenotype. Yasuda et al. reported that inhibitors of the JAK/STAT3 pathway are able to block CAF senescence induced by pro-inflammatory cytokines [61,62]. Moreover, IL-6 knockout markedly attenuated the pro-metastatic effect of sorafenib and increased the susceptibility of liver cancer to the drug through a STAT3-dependent mechanism. In our experiments, we observed a reduction in STAT3 mRNA level following MM-129 treatment in DLD-1 and HT-29 zebrafish xenografts. Simultaneous administration of 5-FU with MM-129 restored pStat3 expression to a level close to that of the control. Additionally, blocking STAT3 via LLL12 significant decreased p21 levels, a key mediator of cellular senescence, below level induced by 5-FU-MM-129 what may indicate the involvement of STAT3 in the senotherapeutic mechanism of MM-129 action. It is worth nothing that nintedanib induces a senolytic effect via STAT3 inhibition [63]. This finding is consistent with ours, as nintedanib suppressed activation of the JAK2/STAT3 pathway, leading to the clearance of drug-induced senescent cells.

Constitutive STAT3 activation is a major driver of tumor initiation, progression, metastasis, and recurrence, all of which are linked to poor clinical outcomes. In colorectal cancer, persistent STAT3 signaling has been implicated in chemoresistance, particularly to 5-FU, whereas its inhibition can restore drug sensitivity. Yue and colleagues demonstrated that STAT3 regulates 5-FU resistance in CRC by promoting Mcl-1-dependent cytoprotective autophagy.

In our study, we observed an elevation in STAT3 expression in DLD-1 and HT-29 cells when 5-FU was administered alone. Conversely, simultaneous use of MM-129 with 5-FU significantly decreased STAT3 expression levels compared to both the control and 5-FU alone, but not compared to MM-129 alone. These results suggest that STAT3 inhibition may be beneficial in overcoming 5-FU resistance, and MM-129 may act as a chemosensitizer. However, further research is needed to confirm these findings.

## 5. Conclusions

Our results highlight the translational relevance of MM-129 as a senotherapeutic candidate. By attenuating chemotherapy-induced senescence-associated features and reducing the burden of senescent cells in colorectal cancer models—including analyses of tumor tissues—this study provides a strong preclinical foundation for “one-two punch” combination strategies. This therapeutic approach involves a sequential two-step process, in which the first “punch” consists of inducing senescence in tumor cells through conventional chemotherapy such as 5-FU. In the “one-two punch” approach, the second step introduces a senotherapeutic agent such as MM-129 to either selectively eliminate senescent cells or suppress their SASP, thereby potentially reducing their pro-tumorigenic and pro-inflammatory effects and improving treatment responses. Notably, resistance to 5-fluorouracil (5-FU) remains a significant clinical challenge in colorectal cancer, often leading to treatment failure and disease recurrence. Therefore, identifying agents such as MM-129 that modulate therapy-induced senescence through senolytic and/or senomorphic mechanisms is of considerable importance for refining current therapeutic strategies and improving patient outcomes.

Importantly, preclinical safety studies have demonstrated that MM-129 possesses a favorable safety profile at the effective antitumor dose of 10 μmol/kg. No serious adverse events or significant changes in hematological, biochemical, hepatic, or renal parameters were observed in rodent or zebrafish models. While higher doses were associated with transient, dose-dependent liver injury, these effects were fully reversible and are consistent with the often reversible hepatotoxicity reported for other chemotherapeutic agents. These findings further support the potential of MM-129 as a safe and well-tolerated candidate for future clinical application in colorectal cancer therapy.

The robust *in vitro* and *in vivo* data presented here—complemented by analyses in human tissue samples—support the advancement of MM-129 towards further preclinical validation and, potentially, early-phase clinical trials in patients with colorectal cancer. Additionally, the mechanisms elucidated here may have broader implications for the development of senotherapeutic approaches in other malignancies.

## Figures and Tables

**Table 1:** Primer sequences used for qPCR analysis.

Gene	Primer Sequence (5′→3′)
SIRT1	Forward: TAGACACGCTGGAACAGGTTGC Reverse: CTCCTCGTACAGCTTCACAGTC
STAT3	Forward: CTTTGAGACCGAGGTGTATCACC Reverse: GGTCAGCATGTTGTACCACAGG
CDH1	Forward: GCCTCCTGAAAAGAGAGTGGAAG Reverse: TGGCAGTGTCTCTCCAAATCCG
IL-6	Forward: AGACAGCCACTCACCTCTTCAG Reverse: TTCTGCCAGTGCCTCTTTGCTG
TNF-α	Forward: CTCTTCTGCCTGCTGCACTTTG Reverse: ATGGGCTACAGGCTTGTCACTC
p21	Forward: AGGTGGACCTGGAGACTCTCAG Reverse: TCCTCTTGGAGAAGATCAGCCG

## Data Availability

The data presented in this study are available on request from the corresponding author. The data are not publicly available due to the large size of raw files and format limitations. Raw and processed data from qPCR, Western Blot, flow cytometry, and histological experiments can be provided upon reasonable request.
